# Effects of administration of histamine and its H_1_, H_2_, and H_3_ receptor antagonists into the primary somatosensory cortex on inflammatory pain in rats 

**Published:** 2014-01

**Authors:** Esmaeal Tamaddonfard, Nasrin Hamzeh-Gooshchi

**Affiliations:** 1 Department of Basic Sciences, Faculty of Veterinary Medicine, Urmia University, Urmia, Iran; 2 Faculty of Veterinary Medicine, Urmia University, Urmia, Iran

**Keywords:** Formalin-induced pain Histamine, Histamine receptor antagonists, Primary somatosensory cortex, Rats

## Abstract

***Objective(s):*** The present study investigated the effects of microinjection of histamine and histamine H_1_, H_2_, and H_3_ receptor antagonists, chlorpheniramine, ranitidine and thioperamide, respectively into the primary somatosensory cortex (PSC) on inflammatory pain.

***Material and Methods:*** Two stainless steel guide canulas were bilaterally implanted into the PSC of anaesthetized rats. Inflammatory pain was induced by subcutaneous (SC) injection of formalin (50 µl, 2.5%) in the ventral surface of right hind paw. Time durations of licking/biting of the injected paw were recorded as a pain measure.

***Results:*** Formalin produced a biphasic pattern of licking/biting of the injected paw. Histamine at doses of 0.5, 1, and 2 µg decreased the intensity of pain. Chlorpheniramine and ranitidine at the same doses of 1 and 4 µg had no effects, whereas thioperamide at a dose of 4 µg suppressed both phases of formalin-induced pain. Pretreatments with chlorpheniramine and ranitidine at the same dose of 4 µg prevented histamine (2 µg)-induced antinociception. Antinociceptive effects were observed when thioperamide at doses of 1 and 4 µg was used with 0.25 and 1 µg of histamine, respectively. The antinociceptive effects induced by histamine (2 µg) and thioperamide (4 µg) were prevented by prior treatment with naloxone (4 µg).

***Conclusion:*** These results indicate that at PSC levels, histamine through post-synaptic H_1_, H_2_, and pre-synaptic H_3_ receptors might be involved in pain modulation. The endogenous opioid system may be involved in histamine- and thioperamide-induced antinociception.

## Introduction

Pain is a multidimensional phenomenon that encompasses sensory-discriminative, affective-motivational, and cognitive-emotional components mediated by different mechanisms and processed in a neural network (1, 2). Recent years have seen progressive unraveling of the neuroanatomical circuits and cellular mechanisms underlying the induction of pain (3, 4). Contrary to the traditional view that the cerebral cortex is not involved in pain perception, multiple cortical areas including the anterior cingulate cortex, the granular insular cortex, the ventrolateral orbital cortex (VLOC), the motor cortex, and the PSC and secondary somatosensory cortex (SSC) have major roles in the representation and modulation of pain (5, 6). Extracellular unit recording techniques have demonstrated that monkey and cat PSC neurons in the deeper lamina encode the intensity of noxious, mechanical, thermal, and chemical stimulation (7). Besides, PSC neurons responded to nociception induced by CO_2_ laser-heat irradiation of the middle part of the tail in rats (8). Recently, it has been reported that formalin injection in the hind paw of rats resulted in metabolic increases in the cortical structures including PSC (9). 

Histamine via its H_1_, H_2_ and H_3_ receptors participates in modulation of pain. Central administration of histamine produced antinociception in the formalin test in mice and rats (10, 11). Co-administration of temelastine (a histamine H_1_ receptor antagonist) and tiotidine (a histamine H_2_ receptor antagonist) with histamine into the periaqueductal gray inhibited the histamine-induced analgesia in the hot plate test in rats (12). Moreover, central injection of ranitidine and thioperamide (a histamine H_3_ receptor antagonist), but not pyrilamine (a histamine H_1_ receptor antagonist), enhanced the nociceptive threshold in a rat model of neuropathic pain (13).

More recently, the involvement of histamine H_1_, H_2_, and H_3_ receptors in the histamine-induced antinociception was reported in the formalin-induced pain at the level of the dentate gyrus in rats (14, 15). 

The present study was aimed at investigating the implication of histaminergic system in pain perception by microinjection of histamine and its H_1_, H_2,_ and H_3_ antagonists into the PSC using formalin test in rats. In addition, we assessed the contribution of the endogenous analgesic opioid system by microinjection of naloxone prior to histamine and thioperamide.

## Materials and Methods

***Animals***

Healthy adult male Wistar rats, weighing 250–280 g, were used in this study. Rats were maintained in groups of six per cage in a light-dark cycle (light on at 07:00 hr) at a controlled ambient temperature (22 ± 0.5°C) with *ad libitum* food and water. Six rats were used for each experiment. All research and animal care procedures were approved by the Veterinary Ethics Committee of the Faculty of Veterinary Medicine of Urmia University and were performed in accordance with the National Institutes of Health Guide for Care and Use of Laboratory Animals.

***Drugs***

Drugs used in the present study included histamine dihydrochloride, chlorpheniramine maleate, ranitidine hydrochloride, thioperamide maleate, and naloxone hydrochloride. The drugs were purchased from Sigma–Aldrich Inc., St Louis, MO, USA. All drugs were dissolved in sterile normal saline 30 min prior to intra-primary somatosensory (intra-PSC) cortex microinjection.

***Surgical procedure***

To deliver the compounds to be tested, rats were bilaterally implanted with two guide cannulas in each PSC. In brief, each rat was anaesthetized with a mixture of ketamine (80 mg/kg) and xylazine (10 mg/kg) injected intraperitoneally (IP), and then placed in a stereotaxic apparatus (Stoelting, Wood Dale, IL, USA). The scalp was incised, and the skull was leveled off around the bregma. Two 24 gauge, 10 mm stainless-steel guide cannulas were bilaterally implanted into the right and left PSCs. The tip of cannulas was aimed at the following coordinates: -0.80 mm posterior to the bregma, 1.8 mm left and right sides of the midline, and 1.2–1.4 mm below the top of the skull (16). The cannulas were then fixed to the skull using three screws and dental acrylic (Acropars, Tehran, Iran). A 10 mm stylet was inserted into each cannula to keep it patent prior to microinjection. All animals were allowed 14 days to recover from surgery.

***Intra-PSC microinjection***

For intra-PSC microinjections of normal saline (control), histamine (0.25, 0.5, 1, and 2 µg), chlorpheniramine, ranitidine, and thioperamide at the same doses of 1 and 4 µg and naloxone at a dose of 4 µg, a 30 gauge, 10 mm injection needle was attached to a 30 cm polyethylene tube fitted to a 1 µl Hamilton syringe. Then the rat was placed on a wooden plate for a period of 15 min, thereafter the stylet was withdrawn, and the injection needle was inserted into the guide cannula. The volume of the drug solution to be injected into each PSC was 0.25 µl, and the injection was slowly made over a period of 60 sec. The injection needle was left in place for a further 60 sec after the completion of the injection to facilitate the diffusion of the drug. Microinjections of chlorpheniramine, ranitidine, thioperamide, and naloxone were performed 10 min before induction of pain, whereas histamine was microinjected 5 min before intraplantar (IPL) injection of formalin. In the case of intra-PSC co-administration of naloxon plus histamine and naloxone plus thioperamide, naloxone was microinjected 2 min before microinjection of histamine and thioperamide. The drug doses used here were calculated according to our previous studies (14, 15, 17–19). 

***Nociceptive testing***

Formalin test was used for induction of pain. Before rats were pain tested, they were placed in a plexiglass observation chamber (30 × 30 × 25 cm) for 30 min on three successive days to minimize stress-activated pain suppressive mechanisms (20). The formalin test was applied as follows: Fifty μl of 2.5% formalin was injected (SC) into the ventral surface of right hind paw using a 30-gauge injection needle; Following formalin injection, the rat was immediately put back in the observation chamber. Nociceptive behaviors including licking/biting of the injected paw were observed with the help of a mirror angled at 45˚ below the observation chamber. Observation of animal’s behavior was made every 5 min and for 60 min, starting after formalin administration (21). Licking/biting behaviors of the injected paw were chosen as measures of pain, because they are supraspinally mediated behaviors (22). The frequency, duration and level of formalin-induced pain behaviors depend on the specific concentration used and the site of injection (23). In the present study, data collected between 0–5 min after formalin injection represented the first (early) phase and data collected between 15–60 min after injection of formalin represented the second (late) phase (21–23). 

***Cannula verification***

At the end of each experiment, 0.25 µl of methylene blue was injected into each side of PSC. Animals were killed with high dose ether, and perfused intracardially with physiological saline followed by 10% formalin solution. Brains were removed and placed in the formalin (10%) solution. At least 3 days later, the brains were sectioned coronally (50-100 µm) and viewed under a loupe to localize the injection site (16). The results obtained from rats with guide cannula outside the PSC (hindlimb region) were eliminated from the data analysis.

***Statistical analysis***

Data obtained from five min blocks, after (IPL) injection of formalin and normal saline, were analyzed using factorial ANOVA followed by Duncan’s test. To evaluate significant differences among intra-PSC treated groups on the first and second phases of formalin-induced pain, one-way analysis of variance (ANOVA) and Duncan's test were applied. In figures, all values are expressed as the mean ± SEM. A value of *P*<0.05 was considered statistically significant.

## Results

**Figure 1:**
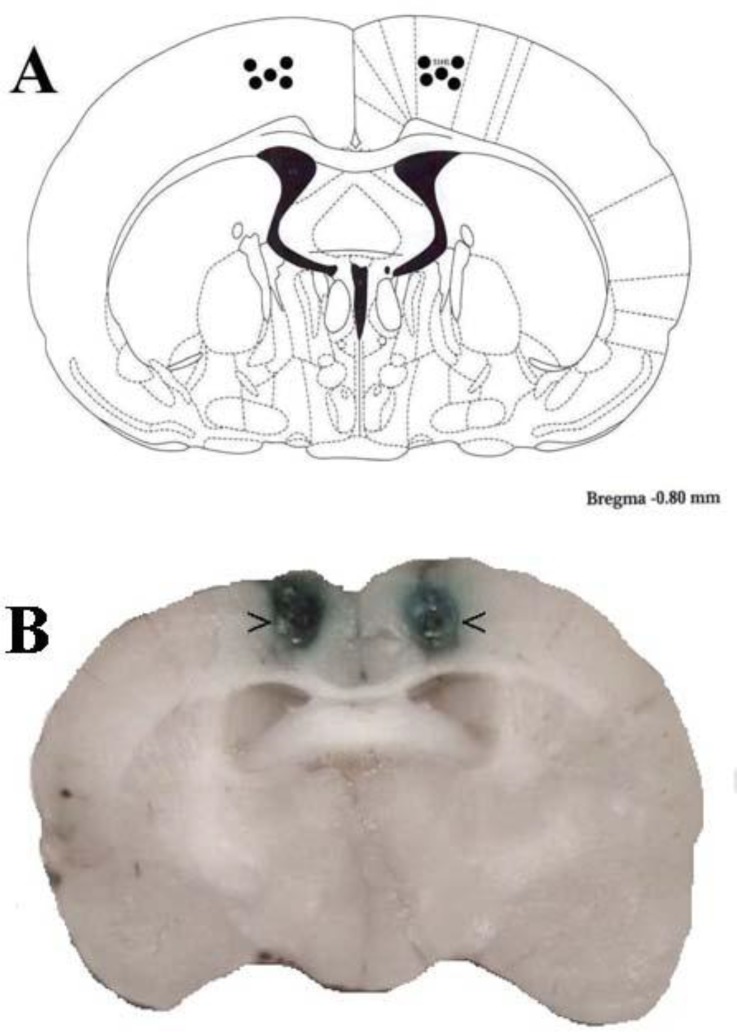
Schematic illustration of coronal section of the rat brain showing the approximate location of the PSC microinjection sites (black circles) in the experiments (A). Locations of the injection cannula tips in the PSC of all rats included in the data analysis (B). Atlas plate adapted from Paxinos and Watson (16)

The placements of the cannula tips in the PSC of rats are shown in Figure 1. The rat brain section was modified from the atlas of Paxinos and Watson (16) (Figure 1A). The locations of the cannula tip placements in the PSC were confirmed with intra-PSC injection of methylene blue (Figure 1B).

**Figure 2:**
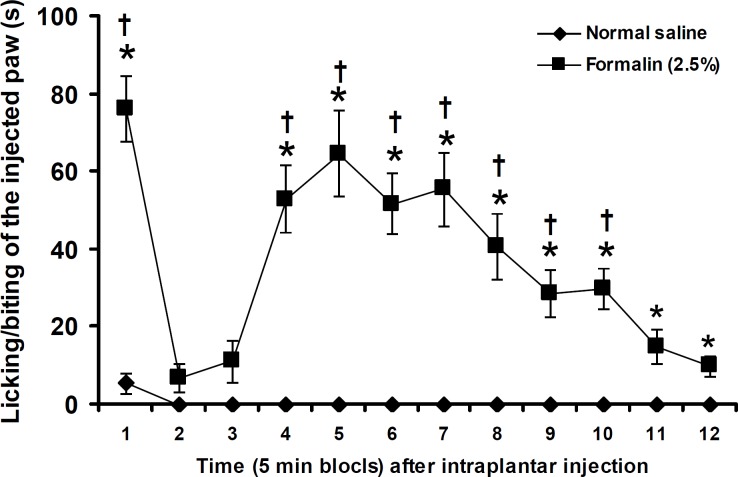
Duration of licking/biting of the hindpaw after IPL injection of normal saline and formalin. Data are presented as mean ± SEM (n = 6). * *P*< 0.05 denotes significant difference *vs* normal saline.^ †^ *P*<0.05 denotes significant difference *vs* other five min blocks (factorial ANOVA and Duncan's test)

Figure 2 shows the durations of licking/biting of normal saline and formalin (2.5%) injected paw at five min blocks. Normal saline produced a negligible response at the first five min block. IPL injection of formalin (2.5%) significantly (*P*<0.05) produced pain responses at 1^st^ and 4^th^–12^th^ five min blocks (Figure 2). 

**Figure 3:**
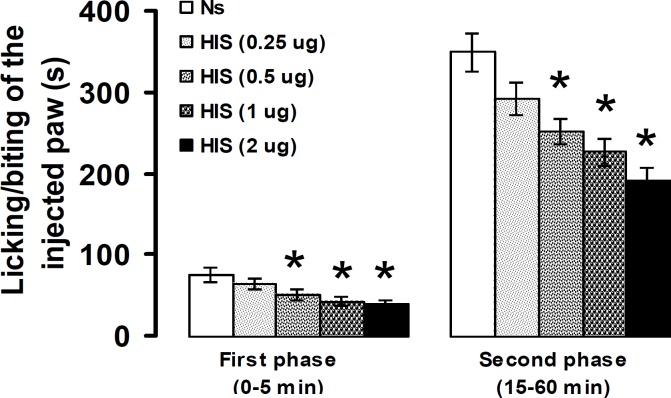
Effect of intra-PSC microinjections of saline normal (Ns) and histamine (HIS) on the formalin-induced pain. Data are presented as mean ± SEM (n = 6). * *P*<0.05 denotes significant difference *vs* normal saline (one-way ANOVA and Duncan's test)

Intra-PSC microinjection of histamine at doses of 0.5, 1, and 2 μg, but not at a dose of 0.25 μg, significantly decreased the intensity of nociceptive response in the first (*P*< 0.05) and second (*P*<0.05) phases of formalin-induced pain (Figure 3). 

**Figure 4:**
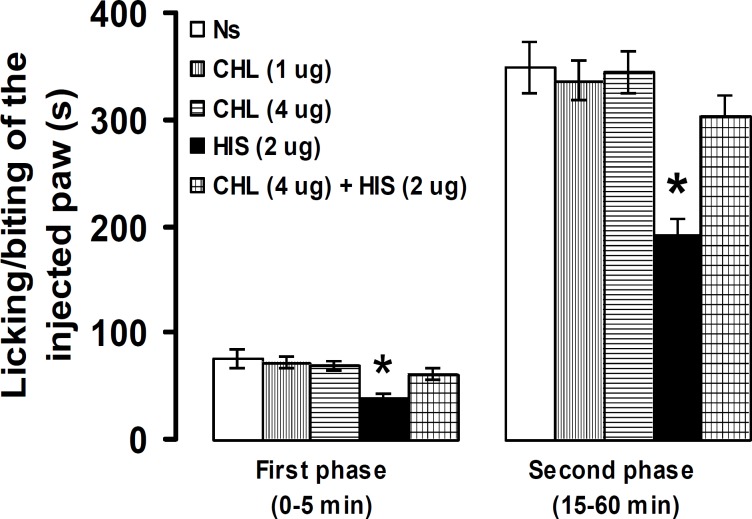
Effect of intra-PSC microinjections of normal saline (NS), chlorpheniramine (CHL) alone and before histamine (HIS) on the formalin-induced pain. Data are presented as mean ± SEM (n = 6). * *P*<0.05 denotes significant difference *vs* other groups (one-way ANOVA and Duncan's test

No significant differences were observed between intra-PSC microinjection of normal saline and chlorpheniramine, at doses of 1 and 4 µg, in pain response. Pretreatment with chlorpheniramine (4 µg) significantly (*P*<0.05) prevented the suppressive effects of histamine (2 µg) on licking/biting of the injected paw in the first and second phases (Figure 4). 

**Figure 5:**
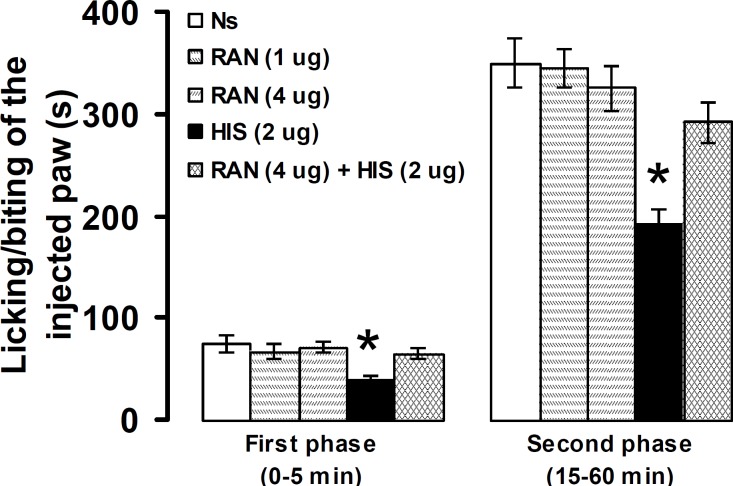
Effect of intra-PSC microinjections of normal saline (Ns), ranitidine (RAN) alone and before histamine (HIS) on the formalin-induced pain. Data are presented as mean ± SEM (n = 6). * *P*<0.05 denotes significant difference *vs* other groups (one-way ANOVA and Duncan's test)

Intra-PSC microinjection of normal saline and ranitidine at doses of 1 and 4 µg produced no significant effects on pain intensity. The suppressive effects, induced by intra-PSC microinjection of histamine (2 µg), on licking/biting of the injected paw in the first and second phases were significantly (*P*<0.05) prevented by prior microinjection of ranitidine (4 µg) into the same site (Figure 5). 

**Figure 6:**
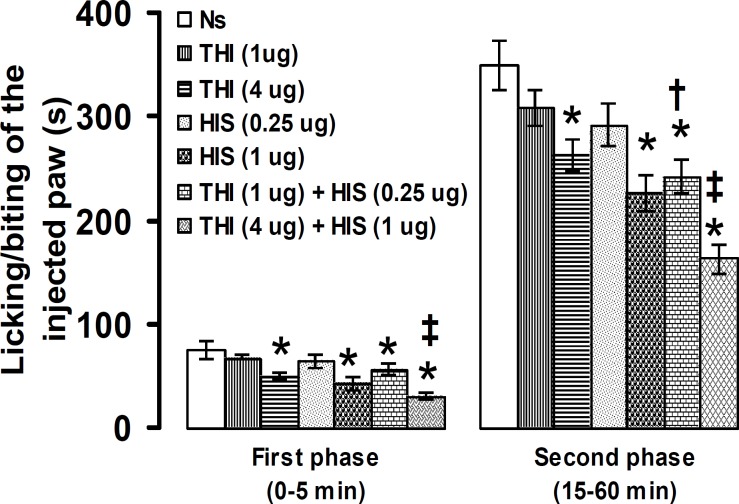
Effect of intra-PSC microinjections of normal saline (Ns), thioperamide (THI) alone and before histamine (HIS) on the formalin-induced pain. Data are presented as mean ± SEM (n = 6). * *P*< 0.05 denotes significant difference *vs* other groups. ^†^ *P*<0.05 denotes significant difference *vs* thioperamide (1 µg) and histamine (0.25 µg). ^‡^ *P*<0.05 denotes significant difference *vs* thioperamide (4 µg) and histamine (1 µg). (one-way ANOVA and Duncan's test)

**Figure 7:**
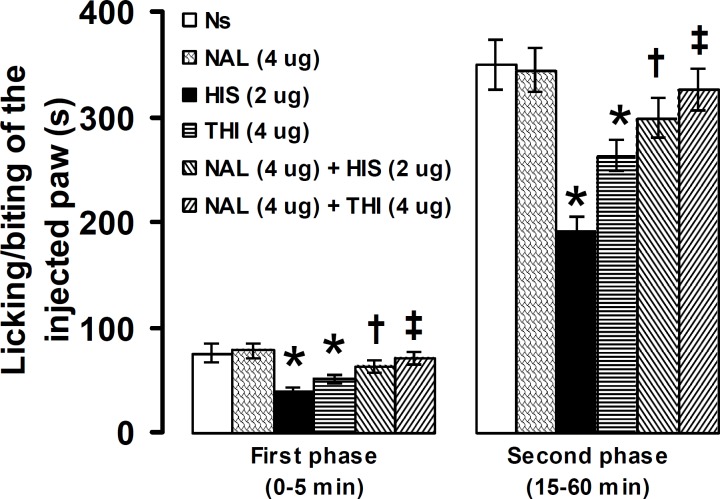
Effect of intra-PSC microinjections of normal saline (Ns), naloxone (NAL) alone and before histamine (HIS), and thioperamide (THI) on the formalin-induced pain. Data are presented as mean ± SEM (n = 6). * *P*<0.05 denotes significant difference *vs* other groups. ^†^ *P*<0.05 denotes significant difference *vs* histamine (2 µg). ^‡^ *P*<0.05 denotes significant difference *vs* thioperamide (4 µg). (one-way ANOVA and Duncan's test)

Microinjection of thioperamide into the PSC at a dose of 4 µg, but not at 1 µg, significantly decreased the duration of licking/biting of the formalin-injected paw in the first and second phases. Thioperamide (1 µg) plus histamine (0.25 µg) significantly (*P*<0.05) decreased the second phase of pain intensity. Thioperamide (4 µg) plus histamine (1 µg) produced antinociception in the first and second phases of formalin-induced pain (Figure 6). 

Intra-PSC microinjection of naloxone (4 µg), significantly (*P*<0.05) prevented the antinociceptive effects induced by histamine (2 µg) and thioperamide (4 µg) in the first and second phases of formalin-induced pain (Figure 7).

## Discussion

The results of the present study showed that IPL injection of formalin produced a biphasic pain response. Formalin test has been used frequently to study pain mechanisms in laboratory animals; according to these studies a biphasic pattern of pain-related behaviors was produced by SC injection of small amounts (20–100 µl) of dilute solutions (0.1–10%) of formalin into the various parts of the body (21–24). The first phase in turn may be attributed to direct algogenic effect of formalin on the nociceptors and the second phase to release of local inflammatory mediators responsible for sensitization of primary and spinal sensory neurons and subsequent signal transduction into the brain (21–24). Various neurotransmitters and nuclei of the brain are involved in the modulation of formalin-induced pain at the supraspinal level (22). 

In the present study, microinjection of histamine into the PSC produced antinociceptive effects in the first and second phases of formalin-induced pain. Histamine H_1_ and H_2_ antagonists, chlorpheniramine and ranitidine respectively, did not alter the intensity of pain when used alone, while pretreatments with chlorpheniramine and ranitidine inhibited histamine-induced antinociception. These indicate that at PSC level, histamine through its post-synaptic H_1_ and H_2_ receptors, modulates formalin-induced pain. Histamine can modulate formalin-induced pain in the peripheral organs, spinal, and supraspinal structures. A small number of flinches were produced due to IPL injection of histamine (25); in addition, IPL injection of chlorpheniramine and cimetidine decreased formalin-induced pain responses in rats (26). At the level of the spinal cord, intrathecal (IT) administration of histamine produced a pain-like behavior consisting of scratching, biting, and licking in conscious mice. IT co-administration of chlorpheniramine or ranitidine with histamine prevented histamine-induced nociceptive behavior (27). At the supraspinal level, it has been reported that co-administration of temelastine or tiotidine with histamine into the periaqueductal gray (PAG) inhibits the histamine-induced analgesia in the hot plate test in rats (28). Moreover, microinjection of mepyramine (a histamine H_1_ receptor antagonist) and ranitidine into the dorsal hippocampus inhibited the suppressive effects of histamine on pain behavior induced by SC injection of formalin in the upper lip region in rats (17). In addition, intra-dentate gyrus microinjections of chlopheniramine and ranitidine prevented histamine-induced antinociception in the paw formalin test in rats (14). PSC has been thought to play an essential role in processing of the sensory-discriminative components of pain. These components include sensing the quality, intensity and spatial location of painful sensations (29). The distribution and function of histamine and its H_1_ and H_2_ receptors in the brain regions including cerebral cortex structures have been reported (30–32). In the cortical structures, histamine H_1_ receptors are distributed in the middle (layers III and IV) and deep layers (layers IV and V), whereas superficial (layers I and II) and middle layer (predominantly layer III) contain histamine H_2_ receptors (32). Using additional factor method and even related potentials recording, it has been found that the histaminergic system via its H_1_ receptors, participates in sensory information processing; and histamine hypofunction in clinical disorders may cause impaired sensory processing (33). Moreover, it has been reported that intravenous (IV) injection of diphenhydramine (a histamine H_1_ receptor antagonist) blocked the inhibitory effect of central amygdaloid nucleus conditioning stimulation on the tooth pulp-driven neuronal activity in the PSC in rats (34). 

In this study, intra-PSC microinjection of thioperamide alone suppressed the pain and produced an antinociceptive effect when co-administered with histamine. This indicates that blockade of histamine H_3_ receptors produced antinociceptive effects. Histamine H_3_ receptors act as pre-synaptic auto-receptors and post-synaptic hetero-receptors (35, 36). Activation of histamine H_3_ auto-receptors by R-α-methylhistamine, imepipe, and imetit (histamine H_3_ receptor agonists) results in the inhibition of histamine synthesis and release from histaminergic neurons, whereas blockade of histamine H_3_ auto-receptors with histamine H_3_ receptor antagonists such as clobenpropit, ciproxifan, and thioperamide can increase the release of histamine from histaminergic endings (37, 38). Recently, Munari *et al* (39) reported that ABT-239, a histamine H3 receptor antagonist/inverse agonist, increased histamine release from the cortex when administered into the TMN of brain in Rats. Although the majority of histamine H_3_ receptors are located in brain (31), histamine H_3_ receptor mRNA is also found in various non-brain tissues including skin, stomach, intestines, brown adipose tissue, dorsal root ganglion, and spinal cord (40, 41). Thus, histamine H_3_ receptors can influence pain modulation at peripheral local, spinal, and supraspinal levels (42). IPL injection of R-α-methylhistamine did not affect thermal hyperalgesia induced by the IPL injection of Complete Freund’s adjuvant in mice (43). In contrast, the activation of spinal histamine H_3_ receptors with IT injection of immepip reduced the first and the second phases of formalin-induced flinching in rats, while the blockade of histamine H_3_ receptors with thioperamide prevented immepip-induced antinociception (44). At the supraspinal level, intracerebroventricular injection of thioperamide and R-α-methylhistamine produced analgesic and hyperalgesic effects, respectively, in the hot plate and writhing tests of nociception in rats and mice (45). Moreover, intra-dentate gyrus microinjection of thioperamide suppressed both the first and the second phases of formalin-induced nociception in rats and increased the antinociceptive effect of histamine when microinjected prior to histamine into the same site. The distribution of histamine H_3_ receptors in the cortical structures (32), may confirm the antinociceptive effect induced by thioperamide that we observed in this study. 

In the present study, naloxone inhibited the antinociceptive effects of histamine and thioperamide on the formalin-induced pain. This means that the opioid receptors may be involved in histamine-induced antinociception. Naloxone, as a competitive antagonist of mu-, kappa-, and sigma-opioid receptors with higher affinity for the mu-opioid receptors (46), has been frequently used to explore the role of endogenous opioid receptors in pain modulation. Microinjections of naloxone into the VLOC reversed the antiociceptive effects of morphine microinjected into the same site in the formalin test and in the L_5_/L_6_ spinal nerve ligation model of neuropathic pain in rats (47, 48). Several interactions exist between histaminergic agents and opioid receptors in modulation of pain. Local activation of histamine H_3_ receptor with IPL injection of R-α-methylhistamine potentiated the suppressive effect of fentanyl in thermal hyperalgesia induced by IPL injection of Complete Ferund’s adjuvant in mice (43). Using the histamine H_3_ receptors gene knockout mice, Mobarakeh *et al* (49) reported that histamine, through its H_3_ receptors exerted inhibitory effects on the antinociceptive effects of morphine at the spinal level. In addition, the microinjection of naloxone into the PAG reversed the antinociceptive effect induced by the microinjection of histamine into the same site (12). The antinociception induced by microinjection of thioperamide into the dentate gyrus was inhibited by prior microinjection of naloxone into the same site (15).

Multiple neurotransmitters including GABA, glutamate, and opioid modulate the pain processing in the cortical structures (6). Both NMDA and AMPA/kainate receptors of glutamate have been found to contribute to PSC high-threshold responses evoked by noxious stimulation of the forepaw or hindpaw in rats (50). Microinjection of GABA_A_ agonist, muscimol, into the PSC significantly reduced the licking behavior in the first and second phases of formalin-induced pain (51). Thus, the neurotransmitters such as glutamate, GABA, opioids, and histamine can modulate pain by influencing the mechanisms of pain perception in the PSC. However, it needs more study with the agents that inhibited or stimulated the release or the synthesis of histamine at the synaptic site to clarify the exact roles of histamine and its receptors on supraspinal modulation of pain. 

## Conclusion

The results of the present study indicate that the activation of brain histamine in the PSC, by exogenous administration of the amine, produced antinociception in the formalin test in rats. The histamine post-synaptic H_1_, H_2,_ and pre-synaptic H_3_ receptors mediate histamine-induced antinociception at the PSC of the brain. Moreover, opioid receptors in the PSC may be involved in the antinociceptive effects of histamine and thioperamide.

## References

[1] Almeida TF, Roizenblatt S, Tufik S (2004). Afferent pain pathways: a neuroanatomical study. Brain Res.

[2] Melzack R (1999). From the gate to the neuromatrix. Pain.

[3] Ossipov MH, Dussor GD, Porreca F (2010). Central modulation of pain. J Clin Invest.

[4] Basbaum AI, Bautista DM, Scherrer G, Julius D (2009). Cellular and molecular mechanisms of pain. Cell.

[5] Ploner M, Schuitzler A (2004). Cortical representation of pain. Nervenarzt.

[6] Xie YF, Huo FQ, Tang JS (2009). Cerebral cortex modulation of pain. Acta Pharmacol Sin.

[7] Kenshalo DR, Iwata K, Sholas M, Thomas DA (2000). Response properties and organization of nociceptive neurons in area 1 of monkey primary somatosensory cortex. J Neurophysiol.

[8] Kuo CC, Yen CT (2005). Comparison of anterior cingulated and primary somatosensory neuronal responses to noxious laser-heat stimuli in conscious, behaving rats. J Neurophysiol.

[9] Shih YYI, Chiang YC, Chen JC, Huang CH, Chen YY, Liu RS (2008). Brain nociceptive imaging in rats using 18F- fluorodeoxyglucose small animal positron emission tomography. Neuroscience.

[10] Tamaddonfard E, Rahimi S (2004). Central effect of histamine and peripheral effect of histidine on the formalin-induced pain response in mice. Clin Exp Pharmacol Physiol.

[11] Mojtahedin A, Tamaddonfard E, Zanbouri A (2008). Antinociception induced by central administration of histamine in the formalin test in rats. Indian J Physiol Pharmacol.

[12] Thoburn KK, Hough LB, Nalwalk JW, Mischler SA (1994). Histamine-induced modulation of nociceptive responses. Pain.

[13] Huang L, Adachi N, Nagaro T, Liu K, Arai T (2007). Histaminergic involvement in neuropathic pain produced by partial ligation of the sciatic nerve in rats. Reg Anesth Pain Med.

[14] Khalilzadeh E, Tamaddonfard E, Farshid AA, Erfanparast A (2010). Microinjection of histamine into the dentate gyrus produces antinociception in the formalin test. Pharmacol Biochem Behav.

[15] Khalilzadeh E, Tamaddonfard E, Farshid AA, Erfanparast A (2010). Thioperamide-induced antinociception is mediated through endogenous opioid system in the dentate gyrus of adult rats. Vet Res Forum.

[16] Paxinos G, Watson C (1997). The rat brain in stereotaxic coordinates.

[17] Erfanparast A, Tamaddonfard E, Farshid AA, Khalilzadeh E (2010). Effect of microinjection of histamine into the dorsal hippocampus on the orofacial formalin-induced pain in rats. Eur J Pharmacol.

[18] Erfanparast A, Tamaddonfard E, Farshid AA, Khalilzadeh E (2010). Antinociceptive effect of morphine microinjections into the dorsal hippocampus in the formalin-induced orofacial pain in rats. Vet Res Forum.

[19] Tamaddonfard E, Erfanparast A, Farshid AA, Khalilzadeh E (2011). Interaction between histamine and morphine at the level of the hippocampus in the formalin-induced orofacial pain in rats. Pharmacol Rep.

[20] Abbott FV, Bonder M (1997). Options for management of acute pain in the rat. Vet Res.

[21] Tjolsen A, Berge OG, Hunskaar S, Rosland GH, Hole K (1992). The formalin test: an evaluation of the method. Pain.

[22] Porro CA, Cavazzuti M (1993). Spatial and temporal aspects of spinal cord and brainstem activation in the formalin pain model. Prog Neurobiol.

[23] Capone F, Aloisi AM (2004). Refinement of pain evaluation techniques. Ann Ist Super Sanita.

[24] Raboisson P, Dallel R (2004). The orofacial formalin test. Neurosci Biobehav Rev.

[25] Hong Y, Abbott FV (1994). Behavioral effects of intraplantar injection of inflammatory mediators in the rat. Neuroscience.

[26] Parada CA, Tambeli CH, Cunha FQ, Ferreira SH (2001). The major role of histamine and 5-hydroxytryptamine in formalin-iduced nociception. Neuroscience.

[27] Sakurada S, Orito T, Furuta S, Watanabe H, Mobarake JI, Yanai K (2003). Intrathecal histamine induces spinally mediated behavioral responses through tachykinin NK1 receptors. Pharmacol Biochem Behav.

[28] Thoburn KK, Hough LB, Nalwalk JW, Mischler SA (1994). Histamine-induced modulation of nociceptive responses. Pain.

[29] Bushnell MC, Duncan CH, Hofbauer RK, Ha B, Chen JI, Carrier B (1999). Pain perception: is there a role for primary sensory cortex?. Proc Nalt Acad Sci USA.

[30] Schwartz JC, Arrang JM, Garbarg M, Pollard H, Rouat M (1991). Histaminergic transmission in the mammalian brain. Physiol Rev.

[31] Pillot C, Heron A, Cochois V, Tardivel-Lacombe J, Ligneau X, Schwartz JC (2002). A detailed mapping of the histamine H3 receptor and its gene transcripts in rat brain. Neuroscience.

[32] Jin CY, Panula P (2005). The laminar histamine receptor system in human prefrontal cortex suggests multiple levels of histaminergic regulation. Neuroscience.

[33] Van Ruitenbeek P, Vermeeren A, Smulders FTY, Sambeth A, Reidel WJ (2009). Histamine H1 receptor blockade predominantly impairs sensory processes in human sensorimotor performance. Br J Pharmacol.

[34] Kawarada K, Kamata K, Matsumoto N (1999). Effects of conditioning stimulation of the central amygdaloid nucleus on tooth pulp-driven neurons in the cat somatosensory cortex (SI). Jpn J Physiol.

[35] Arrang JM, Garbarg M, Lancelot JC, Lecomte JM, Pollard H, Robba M (1987). Highly potent and selective ligands for histamine H3-receptors. Nature.

[36] Pollard H, Moreau J, Arrange JM, Schwartz JC (1993). A detailed authoradiographic mapping of histamine H3 receptors in rat brain areas. Neuroscience.

[37] Brown RE, Stevens DR, Haas HL (2001). The physiology of brain histamine. Prog Neurobiol.

[38] Haas HL, Sergeeva OA, Selbach O (2008). Histamine in the nervous system. Physiol Rev.

[39] Munari L, Provensi G, Passani MB, Blandina, P (2013). Selective brain region activated by histamine H3 receptor antagonist/inverse agonist ABT-239 enhances acetylcholine and histamine release and increased c-Fos expression. Neuropharmacology.

[40] Karlstedt K, Ahman MJ, Anichtchik OV, Soinila S, Panula P (2003). Expression of the H3 receptor in the developing CNS and brown fat suggests novel roles for histamine. Mol Cell Neurosci.

[41] Cannon KE, Chazot PL, Hann V, Shenton F, Hough LB, Rice FL (2007a). Immunohistichemical localization of histamine H3 receptors in rodent skin, dorsal root ganglion, superior cervical ganglion and spinal cord: potential antinociceptive targets. Pain.

[42] Hough LB, Rice FL (2011). H3 receptors and pain modulation: peripheral, spinal and brain interactions. J Pharmacol Exp Ther.

[43] Fernandez-Duenas V, Ciruela F, Gandia J, Sanchez S, Planas E, Poveda R (2010). Histamine H3 receptor activation potentiates peripheral opioid-mediated antinociception: substance P role in peripheral inflammation in mice. Eur J Pharmacol.

[44] Cannon KE, Leurs R, Hough LB (2007b). Activation of peripheral and spinal histamine H3 receptors inhibits formalin-induced inflammation and nociception, respectively. Pharmacol Biochem Behav.

[45] Malmberg-Aiello P, Lamberti C, Ghelardini C, Giotti A, Bartolini A (1994). Role of histamine in rodent antinociception. Br J Pharmacol.

[46] Trescot AM, Datta S, Lee M, Hansen H (2008). Opioid Pharmacology. Pain Physician.

[47] Xie YF, Wang J, Huo FQ, Jia H, Tang JS (2004). Mu but not delta and kappa opioid receptor involvement in ventrolateral orbital cortex opioid-evoked antinociception in formalin test rats. Neuroscience.

[48] Zhao M, Wang JY, Jia H, Tang JS (2006). Mu- but not delta- and kappa-opioid receptors in the ventrolateral orbital cortex mediate opioid-induced antiallodynia in a rat neuropathic pain model. Brain Res.

[49] Mobarakeh JI, Takahashi K, Yanai K (2009). Enhanced morphine-induced antinociception in histamine H3 receptor gene knockout mice. Neuropharmacology.

[50] Pollard M (2000). Ionotropic glutamate receptor-mediated responses in the rat primary somatosensory cortex evoked by noxious and innocuous cutaneous stimulation in vivo. Exp Brain Res.

[51] Wang N, Wang JY, Luo F (2009). Corticofugal output facilitate acute, but inhibit chronic pain in rats. Pain.

